# Transcriptome Analysis Reveals Differential Splicing Events in IPF Lung Tissue

**DOI:** 10.1371/journal.pone.0092111

**Published:** 2014-03-19

**Authors:** Tracy Nance, Kevin S. Smith, Vanessa Anaya, Rhea Richardson, Lawrence Ho, Mauro Pala, Sara Mostafavi, Alexis Battle, Carol Feghali-Bostwick, Glenn Rosen, Stephen B. Montgomery

**Affiliations:** 1 Department of Pathology, Stanford University, Stanford, California, United States of America; 2 Division of Pulmonary and Critical Care Medicine, University of Washington, Seattle, Washington, United States of America; 3 Department of Computer Science, Stanford University, Stanford, California, United States of America; 4 Division of Pulmonary, Allergy, and Critical Care Medicine, University of Pittsburgh School of Medicine, Pittsburgh, Pennsylvania, United States of America; 5 Department of Medicine, Division of Pulmonary and Critical Care Medicine, Stanford University, Stanford, California, United States of America; International Centre for Genetic Engineering and Biotechnology, Italy

## Abstract

Idiopathic pulmonary fibrosis (IPF) is a complex disease in which a multitude of proteins and networks are disrupted. Interrogation of the transcriptome through RNA sequencing (RNA-Seq) enables the determination of genes whose differential expression is most significant in IPF, as well as the detection of alternative splicing events which are not easily observed with traditional microarray experiments. We sequenced messenger RNA from 8 IPF lung samples and 7 healthy controls on an Illumina HiSeq 2000, and found evidence for substantial differential gene expression and differential splicing. 873 genes were differentially expressed in IPF (FDR<5%), and 440 unique genes had significant differential splicing events in at least one exonic region (FDR<5%). We used qPCR to validate the differential exon usage in the second and third most significant exonic regions, in the genes COL6A3 (RNA-Seq adjusted pval = 7.18e-10) and POSTN (RNA-Seq adjusted pval = 2.06e-09), which encode the extracellular matrix proteins collagen alpha-3(VI) and periostin. The increased gene-level expression of periostin has been associated with IPF and its clinical progression, but its differential splicing has not been studied in the context of this disease. Our results suggest that alternative splicing of these and other genes may be involved in the pathogenesis of IPF. We have developed an interactive web application which allows users to explore the results of our RNA-Seq experiment, as well as those of two previously published microarray experiments, and we hope that this will serve as a resource for future investigations of gene regulation in IPF.

## Introduction

Idiopathic pulmonary fibrosis (IPF) is a progressive disease of unknown aetiology, characterized by fibrotic scarring in the lungs which leads to shortness of breath and eventual respiratory failure. The disease typically presents in patients 50–70 years old, with prevalence increasing with age, and has been shown to have both genetic and environmental predisposing factors [1], [2]. Median survival time after diagnosis is only 4–5 years [3], and there is currently no effective treatment for IPF except lung transplantation [4].

Current theory of pathogenesis in IPF holds that chronic injury to alveolar epithelial cells induces aberrant activation of wound-healing pathways, leading to an increase in inflammatory signals and subsequent differentiation of fibroblasts, epilthelial-mesenchymal transition in alveolar cells, and accumulation of myofibroblasts. This results in the formation of fibroblastic foci and deposition of collagen, fibronectin, and other extracellular matrix (EM) components. In contrast with normal wound-healing and for unknown reasons, apoptosis is not properly initiated in myofibroblasts, and secretion of EM proteins does not terminate. This results in contraction and ultimately destruction of the lung parenchyma [3], [4].

The primary cause of alveolar injury and dysregulated repair is still poorly understood, but recent genome-wide association studies have implicated abnormalities in mucosal defense, cell-cell adhesion and DNA repair in the development of IPF [5]. Previous studies have indicated that many other pathways are perturbed in IPF as well, including TGF-*β* and WNT signaling and others related to coagulation, angiogenesis, oxidative stress, and development [4]. Genes associated with these pathways have been found to have differential expression in IPF cases as compared to healthy controls; however, no effective treatment has yet been developed which targets any individual gene.

The ability to interrogate mRNA transcripts through RNA sequencing allows us to find genes whose differential expression reaches genome-wide significance, and to investigate differential splicing events on a broad scale. Furthermore this transcriptome-wide information can be used to inform the study of pathways and networks which may be dysregulated in IPF. We performed RNA sequencing on whole lung tissue samples obtained from 8 patients with IPF and 7 healthy controls in order to investigate these phenomena and their potential role in the pathogenesis of this complex disease.

In addition, a large number of microarray-based gene expression studies of IPF have been published and the data are publicly accessible, but there is currently no easy way to visualize differential expression results across various studies. We further wanted to make splicing visualizations from our study easily available and searchable. With this in mind, we have developed an internet application which allows users to view the results of our RNA-Seq study interactively, and to compare them with results of previously published microarray studies: The IPF Gene Explorer, available from the project website link at http://montgomerylab.stanford.edu/resources.html. We hope that this application will make gene expression results more accessible to researchers and will be a valuable tool in future investigations.

## Results

### Differential Gene Expression

**Figure 1:**
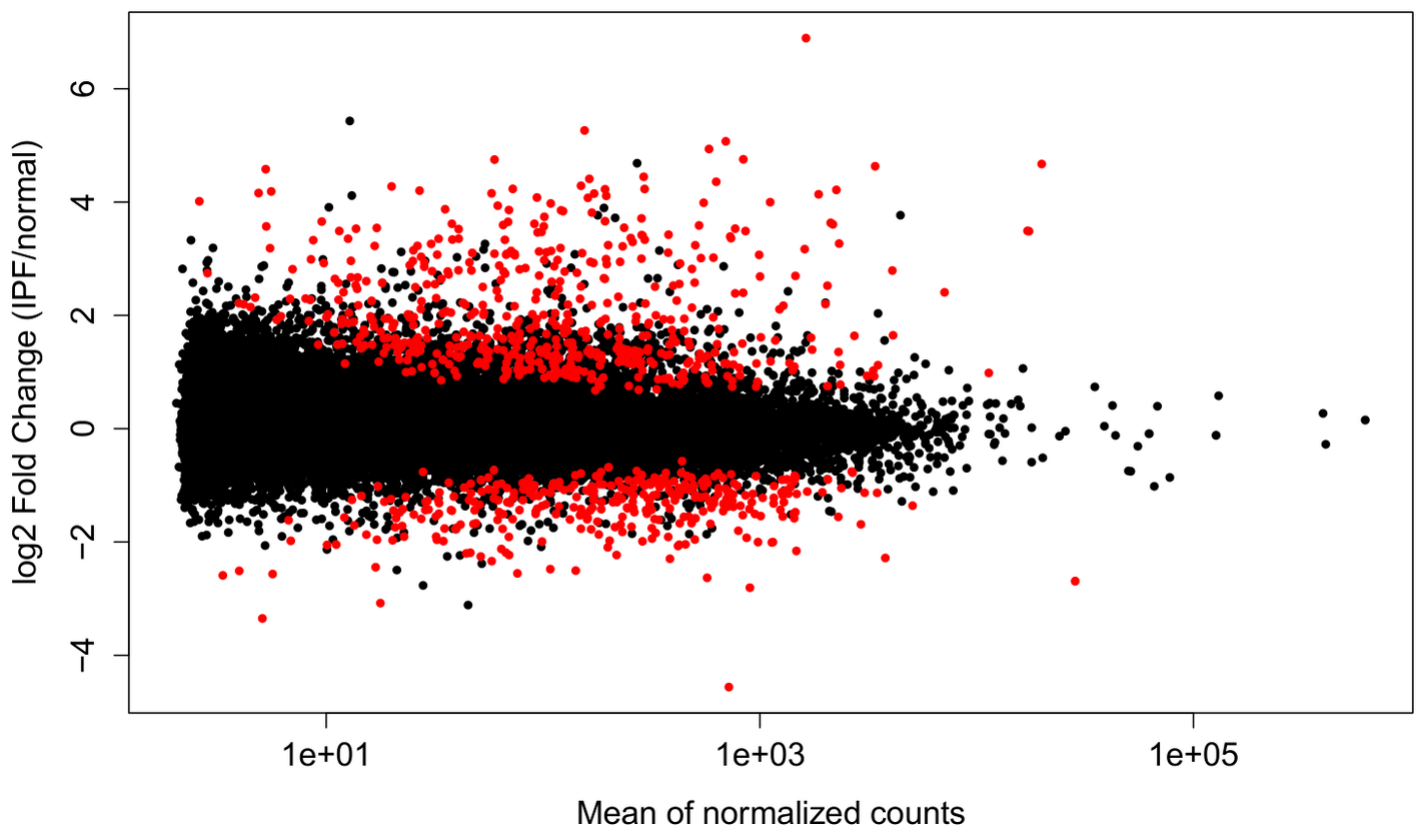
Differential expression analysis reveals more upregulation than down regulation. This plot depicts fold change vs. mean expression. Points depict genes, with red indicating those genes that show significant differential expression (FDR<5%).

In comparing the healthy (n = 7) and diseased (n = 8) lung samples, the Bioconductor package DESeq [6] produced a list of 873 genes showing significant differential expression (DE) at an FDR of 5%, where sex and demographic group were included as covariates. These genes had fold changes in the range
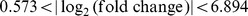
with one gene (LGALS7) having no counts in the healthy samples. Overall, we observed more up-regulated genes in the IPF samples than down-regulated genes (Figure 1). The top ten genes with smallest p-value are listed in Table 1.

**Table 1:** Top ten differentially expressed genes (by p-value).

gene	pval	padj	log2FC	description
COMP	4.17e-20	1.28e-15	3.46	cartilage oligomeric matrix protein
DIO2	1.35e-18	2.08e-14	2.75	deiodinase, iodothyronine, type II
CXCL14	1.19e-15	1.22e-11	4.11	chemokine (C-X-C motif) ligand 14
IGLC3	6.04e-15	4.65e-11	4.14	immunoglobulin lambda constant 3 (Kern-Oz+ marker)
PDGFD	1.18e-14	7.29e-11	2.51	platelet derived growth factor D
MMP13	4.11e-14	2.11e-10	3.52	matrix metallopeptidase 13 (collagenase 3)
CDH3	6.83e-14	3.01e-10	2.00	cadherin 3, type 1, P-cadherin (placental)
RP11-731F5.2	3.17e-13	1.22e-09	4.36	lincRNA
IGHG2	4.96e-13	1.70e-09	4.21	immunoglobulin heavy constant gamma 2 (G2m marker)
IGHGP	1.26e-12	3.87e-09	4.41	immunoglobulin heavy constant gamma P (non-functional)

There were 82 unique named genes which showed highly significant DE (FDR<1%) in both our RNA-Seq data and in two recent microarray studies [7], [8]. At an FDR of <1%, our study identified 475 differentially expressed (DE) genes (8 IPF samples and 7 controls), whereas microarray experiment GSE24206 (17 IPF, 6 controls) identified 3083 DE genes and microarray experiment GSE32537 (119 Idiopathic Interstitial Pneumonias, 50 controls) identified 6291 DE genes. The direction of change found in the RNA-Seq data agrees with that from both microarray studies for all 82 genes in this overlap. A list of these genes, together with their p-values and fold changes, may be viewed on the “Gene Set” page of our web application, by selecting the gene set “Microarray overlap” and clicking on the “Table” tab.

### GWAS Genes are Enriched for Differential Expression

198 SNPs were retrieved from the discovery set of a recent GWAS study [5] of idiopathic interstitial pneumonia (IIP), a class of diseases which includes IPF and similar fibrotic diseases of the lung. The discovery SNPs were those having GWAS pvalue <0.0001. We identified 111 genes which were associated with these SNPs via biomaRt [9] and for which we had sufficient read depth to test for DE in our RNA-Seq data; of these genes, 8 showed significant differential expression at an FDR of 5% and are listed in Table 2. We calculated a hypergeometric p-value of 0.0132 for the likelihood of seeing 8 significant hits in a gene set of this size (see Methods), indicating that it is unlikely to see this many genes showing differential expression by chance alone. Because questions have been raised about the validity of the hypergeometric (or Fisher’s exact) test in this context [10], we also calculated an empirical p-value of 0.01 by a sample permutation method.

**Table 2:** Genes associated with GWAS [5] validation SNPs which are differentially expressed in RNA-Seq data at 5% FDR.

gene	pval	padj	log2FC	description	GWAS significant rsids
RNF5	9.46e-07	2.04e-04	−2.16	ring finger protein 5, E3 ubiquitin protein ligase	rs3134943
**MUC5B**	**6.50e-06**	**9.05e-04**	**4.63**	**mucin 5B, oligomeric mucus/gel-forming**	**rs12417955, rs2735727, rs2857476, rs868903**
**DSP**	**8.83e-05**	**6.40e-03**	**1.16**	**desmoplakin**	**rs10484325, rs10484326, rs2076295, rs2076302, rs3778337**
AGER	1.03e-04	7.27e-03	−2.28	advanced glycosylation end product-specific receptor	rs3134943
SRGAP3	2.45e-04	1.41e-02	1.31	SLIT-ROBO Rho GTPase activating protein 3	rs12638703
MAPK10	7.46e-04	3.23e-02	0.76	mitogen-activated protein kinase 10	rs4488910
FAT1	9.27e-04	3.71e-02	0.93	FAT atypical cadherin 1	rs2130910
HBEGF	1.33e-03	4.76e-02	−1.05	heparin-binding EGF-like growth factor	rs13385

The two genes in bold have corresponding SNPs which were validated by the GWAS meta-analysis.

**Figure 2:**
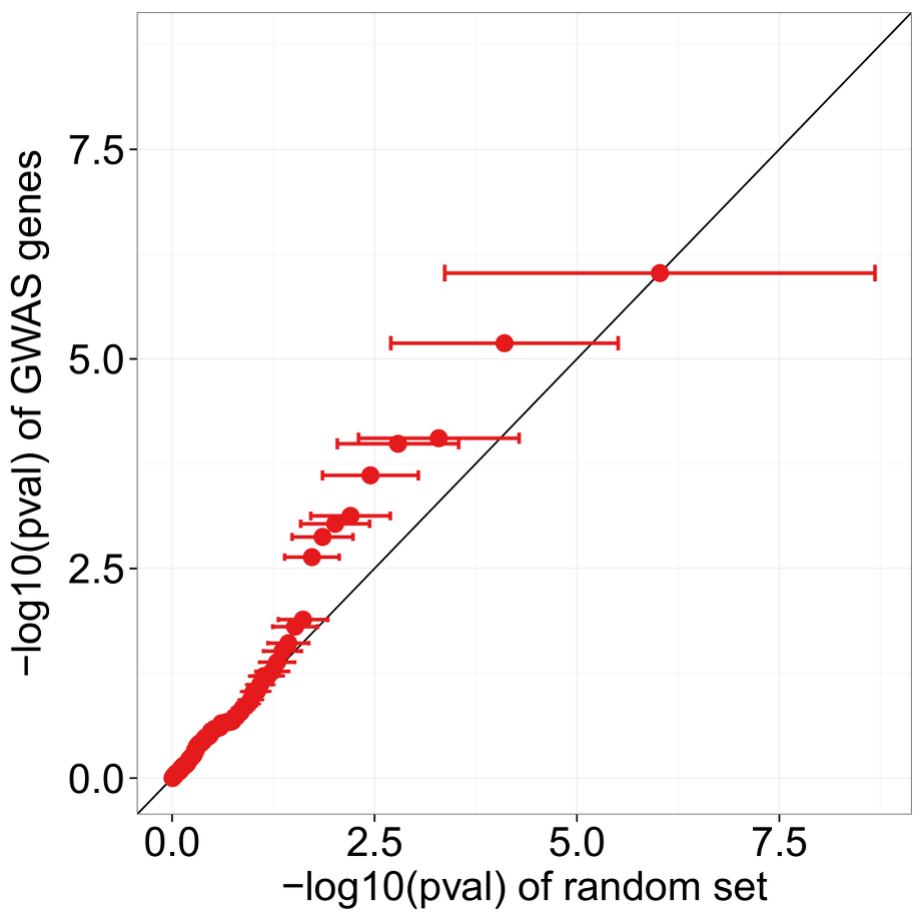
GWAS genes identified from discovery SNPs appear to be enriched for differential expression. *X*-axis values of dots indicate the mean −log10(pval) from 10,000 randomly permuted sets, and error bars indicate one standard deviation from the mean in these permutations.

The definition of significant differential expression above relies on an arbitrary FDR cutoff, but we similarly found enrichment for differential expression using FDR cutoffs of 1% (p-value = 0.093) and 10% (p-value = 0.040). An alternative view is given by the QQ-plot in Figure 2, which depicts the quantiles of −log_10_ p-values for the 111 GWAS-identified genes versus the means of quantiles of −log_10_ p-values across 10,000 random gene sets, with error bars depicting one standard deviation from the mean. This plot suggests that the GWAS genes are enriched for more significant DE p-values, even among those that do not pass our 5% FDR cutoff for significance.

**Figure 3:**
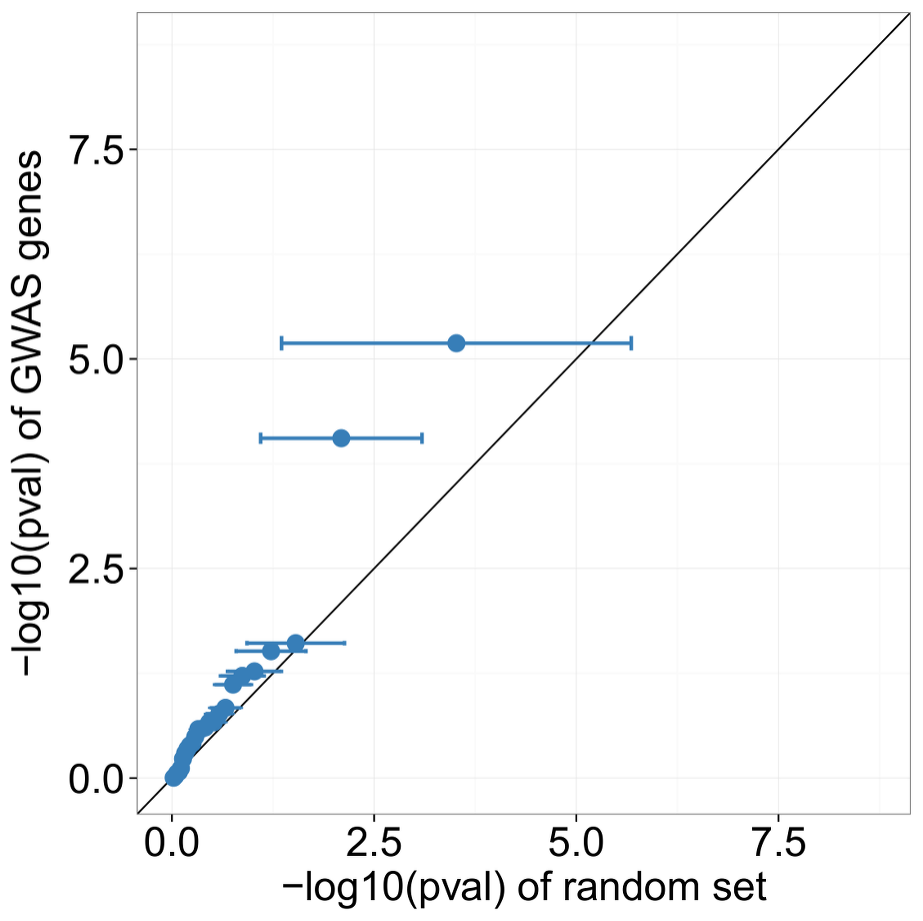
GWAS genes identified from validated SNPs appear to be enriched for differential expression. *X*-axis values of dots indicate the mean −log10(pval) from 10,000 randomly permuted sets, and error bars indicate one standard deviation from the mean in these permutations.

**Figure 4:**
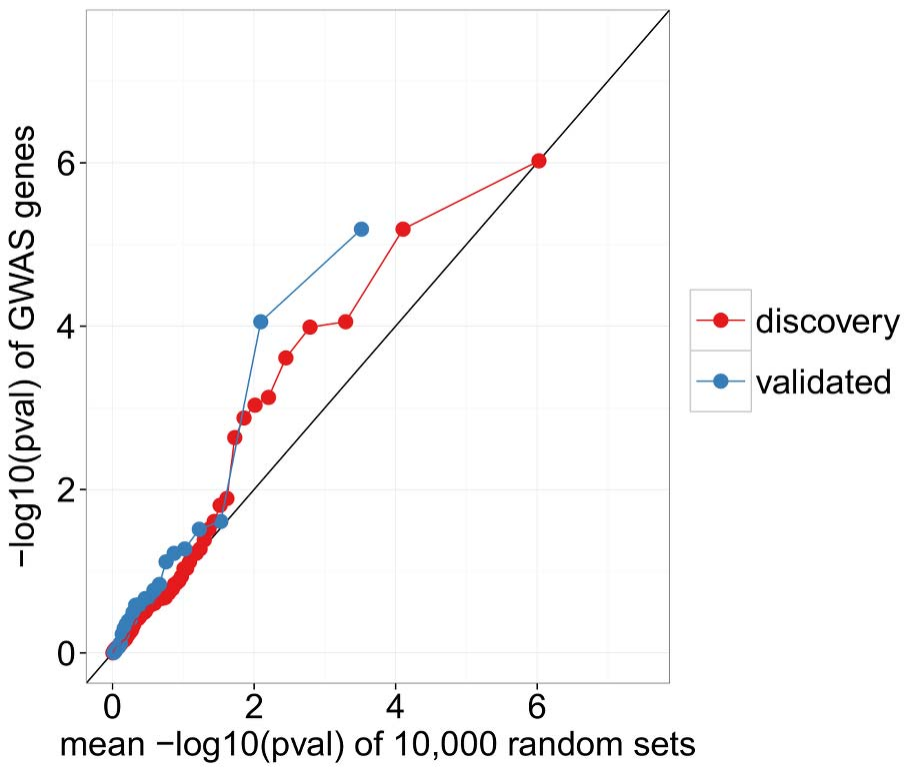
GWAS identified genes appear to be enriched for differential expression. We see smaller differential expression p-values for GWAS genes than for randomly selected genes.

For the 45 SNPs identified as being significantly associated with IIP in the meta-analysis of [5], 25 corresponded to genes we tested in DESeq with 2 being significant, giving a hypergeometric p-value of 0.158. Although this p-value is not as low as the p-value for the discovery set of GWAS SNPs, the QQ-plots in Figure 3 and Figure 4 show that the distribution of p-values in validated GWAS SNPs has a larger deviation from the diagonal, indicating that these higher confidence GWAS SNPs may still have a greater enrichment for DE genes than the discovery set.

The most striking observation about the genes in Table 2 is that all have been implicated in cellular adhesion, migration, or invasion, underscoring the observation made in [5] that cell-cell adhesion should be high on the list of processes considered for further research and potential therapeutic interventions.

Our results confirm those of [5] showing that MUC5B and DSP are significantly over-expressed in IPF lungs as compared to controls, and these genes are discussed in detail in this previous study; in particular DSP is involved in cell adhesion via the connection of cytoskeleton to cell membrane.

RNF5 is an E3 ubiquitin ligase which has been associated with focal adhesion as well as endoplasmic reticulum stress response [11], and is proposed to be a regulator of breast cancer progression through its effects on actin cytoskeleta [12]. RNF5 acts by ubiquitination of the protein paxillin, decreasing its localization in focal adhesions and impairing cell motility [13].

AGER encodes a receptor for advanced glycosylation end-product which is normally highly expressed in adult lung, and which has been shown to be significantly down-regulated in IPF lungs as compared to controls [14], [15]. A decrease in this gene’s protein product (RAGE) has been shown to impede cellular adhesion to collagen, leading to increased migration of epithelial cells and fibroblasts [15]. RAGE is also associated with the differentiation of alveolar epithelial type II cells into alveolar type I cells [16].

SRGAP3 (MEGAP) is a small GTPase involved in the Slit-Robo pathway which plays a role in neuronal development and has been implicated in X-specific mental retardation [17]. In this context it has been shown that SRGAP3 inhibits the formation of focal complexes and alters the actin and microtubule cytoskeleton, thereby impeding cell migration [18].

MAPK10 is the gene encoding the enzyme mitogen-activated protein kinase 10, also known as JNK3. The related kinase JNK1 contributes to TGF*β*1-induced epithelial to mesenchymal transition and collagen deposition, and has been shown to be necessary for the development of pulmonary fibrosis in a mouse model [19]; however contrary to our findings, this study reports that JNK3 expression is limited to heart, testis, and brain. JNK has been shown to be activated in IPF tissues [20], where it appears to play a role in persistence of myofibroblasts [21], and inhibition of JNK has been shown to enhance cell-cell adhesion [22].

FAT1 encodes a protein that belongs to the cadherin superfamily of membrane proteins. Previous research has shown that FAT1 is involved with actin dynamics and cellular polarization in mammalian (rat/mouse) cell lines; in particular it was shown to be necessary for regulation of cellular motility in wound closing, in that a knockdown of this protein led to a decrease in cell migration [23]. FAT1 has also been associated with liver fibrosis [24].

Heparin-binding EGF-like growth factor (HBEGF) is an epidermal growth factor which affects multiple cell types, including fibroblasts, keratinocytes, and vascular smooth muscle cells, and has been associated in various ways with fibrosis occuring in the heart, liver, and pancreas of mice or rats [25], [26], [27]. It promotes the survival and proliferation of mesenchymal cells like fibroblasts and myofibroblasts, which is a key element in the progression of a normal response to lung injury to a pathologic state of fibrosis [28]. HBEGF has been shown to enhance cellular adhesion to the extracellular matrix, as well as invasion, angiogenesis and EMT in the context of ovarian cancer [29]. It has also been shown to increase migration in keratinocytes [30] and human peritoneal membrane cells [31].

### Network Analysis

The results of our network analysis recapitulate those described in other studies, underscoring that IPF is a disease in which many pathways are disrupted. Tables 3, 4, and 5 list those pathways identified by SPIA [32] as being significantly perturbed at an FDR of <10%, where we provided SPIA with a list of genes found to be significantly differentially expressed in our RNA-Seq data at FDRs of 1%, 5% and 10%, respectively.

**Table 3:** SPIA results when provided with DE genes significant at 1% FDR.

Name	FDR	Status
Salivary secretion	0.0329	Inhibited
Pancreatic secretion	0.0441	Inhibited
ECM-receptor interaction	0.0441	Activated
Focal adhesion	0.0785	Activated

**Table 4:** SPIA results when provided with DE genes significant at 5% FDR.

Name	FDR	Status
Salivary secretion	0.0007	Inhibited
TGF-beta signaling pathway	0.0022	Activated
Amoebiasis	0.0483	Inhibited
ECM-receptor interaction	0.0483	Activated
Basal cell carcinoma	0.0643	Inhibited
Wnt signaling pathway	0.0866	Inhibited

**Table 5:** SPIA results when provided with DE genes significant at 10% FDR.

Name	FDR	Status
Amoebiasis	0.0008	Inhibited
Salivary secretion	0.0008	Inhibited
TGF-beta signaling pathway	0.0035	Activated
Complement and coagulation cascades	0.0254	Inhibited
ECM-receptor interaction	0.0523	Activated
Arrhythmogenic right ventricular cardiomyopathy (ARVC)	0.0523	Activated
Pathways in cancer	0.0523	Inhibited
Dilated cardiomyopathy	0.0676	Activated
Wnt signaling pathway	0.0729	Inhibited
Transcriptional misregulation in cancer	0.0729	Inhibited
Basal cell carcinoma	0.0729	Inhibited

We also performed a gene-set enrichment analysis (see Methods) on the results of the differential expression analyses for our RNA-Seq data, first for gene-level expression and then for differential exon usage. Five pathways showed up as being significant at FDR 10% in both analyses; these are listed in Table 6.

**Table 6:** Pathways enriched for differentially expressed genes and for differentially spliced genes in the RNA-Seq data.

pathway	DE padj	DEX padj
KEGG: ECM-receptor interaction	<0.00275	0.010267
Reactome: Steroid metabolism	<0.00275	0.076444
Reactome: Integrin cell-surface interactions	0.018419	0.083849
Reactome: Signaling by VEGF	0.069841	0.023158
Reactome: Hemostasis	0.095437	0.017600

### Alternative Splicing

The Bioconductor package DEXSeq [33] discovered 675 differentially expressed exonic regions (FDR<5%), which lie in 436 unique named genes. The top ten significant regions are listed in Table 7. We were unable to perform PCR validation for the first exonic hit, RPS24, because the GC-content of the region prevented us from designing a high-quality primer within the differentially expressed exon. However we did validate differential usage in the second and third most significant regions, within the genes POSTN and COL6A3, by using a second exon in each respective gene as a control and proxy for gene-level expression. PCR confirmed that usage of these exons was depressed in IPF as compared to controls, with both exons giving a Wilcoxon rank-sum p-value of 3.108e-4, the smallest p-value possible in this nonparametric rank-based test.

**Table 7:** Top ten differentially expressed exons (by p-value).

gene	pval	padj	exon	description	in IPF
RPS24	1.67e-15	3.42e-10	chr10∶79800373–79800428	ribosomal protein S24	down-regulated
COL6A3	6.99e-15	7.18e-10	chr2∶238296225–238296720	collagen, type VI, alpha 3	down-regulated
POSTN	3.01e-14	2.06e-09	chr13∶38143437–38143520	periostin, osteoblast specific factor	down-regulated
DLC1	5.82e-12	2.99e-07	chr8∶13356583–13357330	deleted in liver cancer 1	up-regulated
COL3A1	4.03e-11	1.66e-06	chr2∶189867756–189867788	collagen, type III, alpha 1	down-regulated
ZFP36L1	4.97e-11	1.70e-06	chr14∶69261464–69262759	ZFP36 ring finger protein-like 1	down-regulated
COL3A1	5.84e-10	1.66e-05	chr2∶189868460–189868513	collagen, type III, alpha 1	down-regulated
GM2A	6.46e-10	1.66e-05	chr5∶150646857–150650001	GM2 ganglioside activator	up-regulated
COL3A1	9.89e-10	2.26e-05	chr2∶189868149–189868190	collagen, type III, alpha 1	down-regulated
TRA2B	1.75e-09	3.30e-05	chr3∶185652294–185654025	transformer 2 beta homolog (Drosophila)	down-regulated

#### POSTN

**Figure 5:**
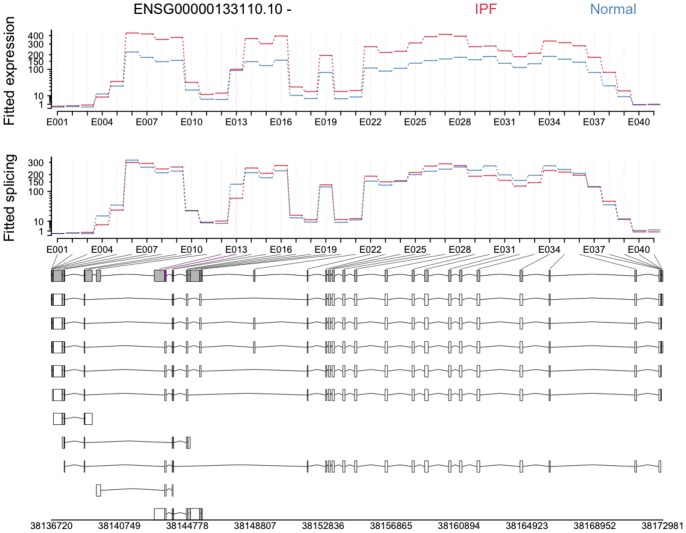
Periostin (POSTN) shows evidence of differential splicing at exon 21. In this splicing plot produced by DEXSeq, exon 21 (labelled E013) is highlighted in magenta.

The third most significant result lies in the gene encoding periostin (POSTN). Figure 5 shows that exon 21, labelled as bin E013 in the figure, is more likely to be spliced out in IPF samples than in controls; shown are the fitted expression and fitted splicing coefficients, for which E013 is relatively lower in IPF than other exonic regions in the gene (see [33] for more on fitted coefficients). To perform real-time PCR validation, we designed primers to amplify exon 21 as well as exon 20, an adjacent exon that is present in all annotated transcripts of POSTN. For this reason, the expression of exon 20 was used as an internal baseline from which to estimate the proportion of POSTN transcripts lacking exon 21.

**Figure 6:**
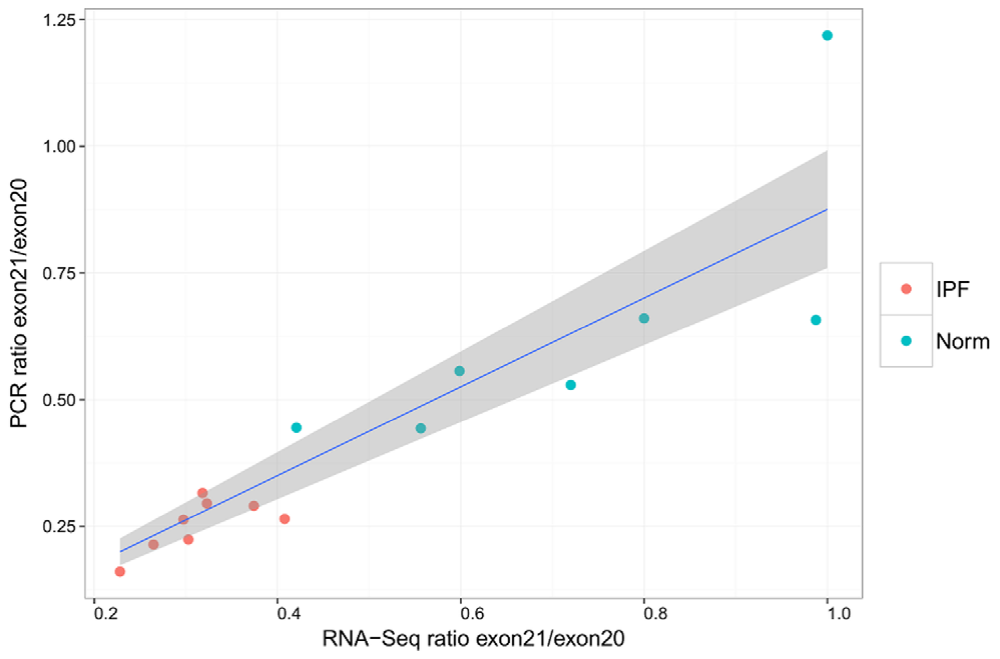
Ratios of periostin exon usage calculated from qPCR vs. RNA-Seq. The grey area indicates 95% confidence interval for the linear regression.

**Figure 7:**
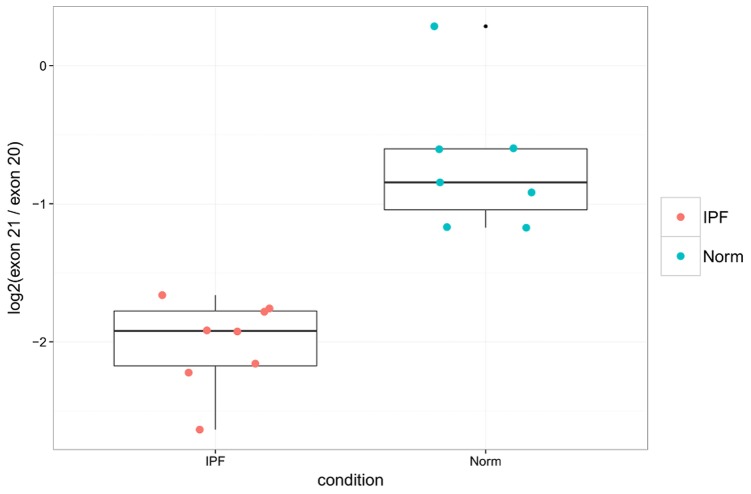
qPCR confirms down-regulation of POSTN exon 21 in IPF lung tissue. Shown are PCR ratios of POSTN exon usage for IPF vs controls (Wilcoxon p-value = 3.108e-4).

The ratios calculated for the expression of exon 21 versus exon 20 by PCR are in good linear agreement with the ratios calculated from normalized counts in the RNA-Seq data: a linear regression with intercept 0 has adjusted *R* ^2^ = 0.946, *F* = 261.9 with p-value 1.86e-10 (Figure 6). In addition the PCR data validates the finding that these ratios are smaller in IPF samples than in healthy controls (Wilcoxon rank-sum p-value = 3.108e-4, Figure 7).

POSTN encodes the secreted extracellular matrix protein periostin, whose increased expression has previously been associated with IPF [34], and whose expression levels have been reported to be predictive of clinical progression in IPF [35]. Other studies have implicated periostin in murine models of lung fibrosis, where it has been shown to induce chemokines to recruit neutrophils and macrophages [36]. Like the proteins associated with GWAS SNPs in the previous section, periostin is involved in cell adhesion and migration. It has been implicated in tumor invasion via a contribution to epithelieal-mesenchymal transition [37], [38], and it enhances TGF-*β*-induced myofibroblast differentiation in neonatal lungs [39].

Although our RNA-Seq data does indicate that gene-level expression of POSTN is increased in IPF samples by a factor of 2, this increase did not reach genome-wide significance in our study (p-value = 9.5e-3), adjusted p-value = 0.1685), whereas relative down-regulation of exon 21 was highly significant (p-value = 3.01e-14, adjusted p-value = 2.06e-9).

Full length periostin consists of 23 exons and there are nine reported isoforms [40]; some of these isoforms were detected only in fetal lung or renal tissue and not in adult lungs. The N-terminal region of the protein is conserved. Isoforms vary in the cassette exons 17–21 of the C-terminal region, and it has been proposed that this region binds ECM proteins like collagen and fibronectin, so that differences in the constitutive exons of this region should affect interactions with the ECM [41]. Indeed it has been shown that periostin isoforms have differential effects on cell invasiveness in several models [42].

#### COL6A3

**Figure 8:**
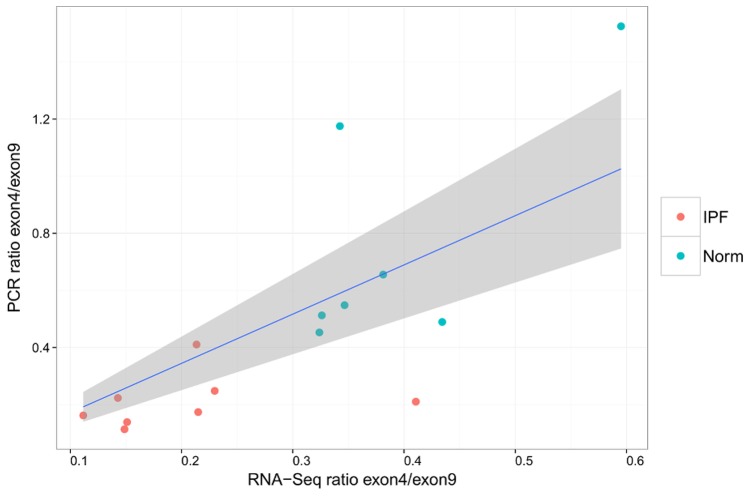
Ratios of COL6A3 exon usage calculated from qPCR vs. RNA-Seq. The grey area indicates 95% confidence interval for the linear regression.

**Figure 9:**
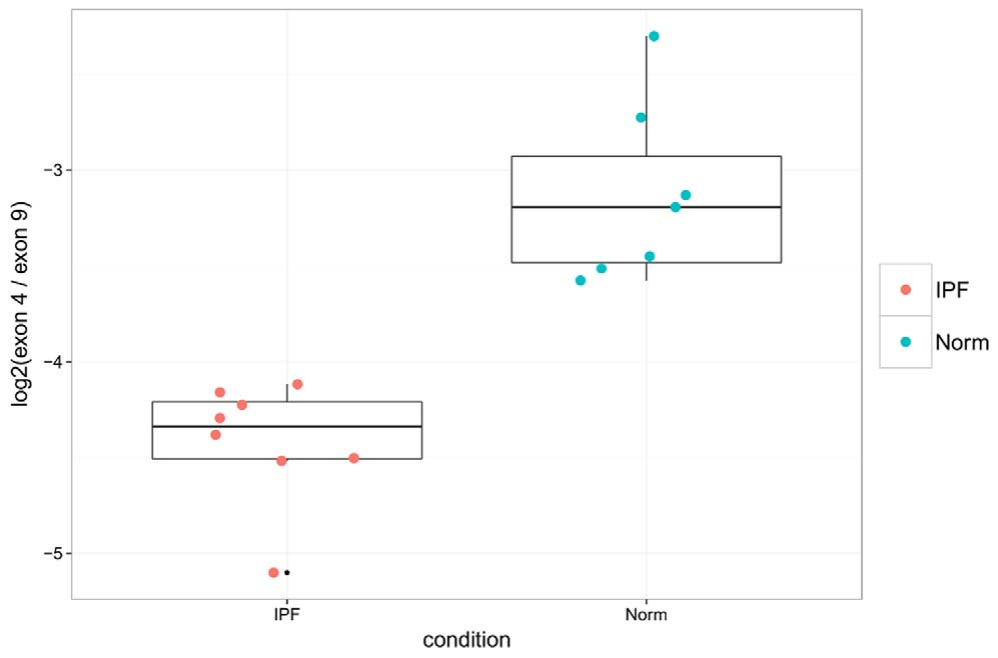
qPCR confirms down-regulation of COL6A3 exon 4 in IPF lung tissue. Shown are PCR ratios of COL6A3 exon usage for IPF vs controls (Wilcoxon p-value = 3.108e-4).

The second most significant result lies in exon 4 of the gene COL6A3, and is more likely to be spliced out in IPF samples than in controls. A probe within exon 9 of COL6A3, which did not show differential splicing in the RNA-Seq data or evidence of SNPs within our samples, was chosen as an internal reference. Ratios calculated for the expression of exon 4 to exon 9 by PCR agree linearly with those calculated by RNA-Seq, giving adjusted *R* ^2^ = 0.803 and *F* = 62.24 with p-value 1.61e-6, calculated as for POSTN (Figure 8). Furthermore the PCR data validates that these ratios are smaller in IPF samples than in healthy controls (Wilcoxon rank-sum p-value = 3.108e-4), as shown in Figure 9).

COL6A3 does not show differential gene-level expression in our RNA-Seq data, but it is significantly upregulated in IPF in the two previously published microarray experiments described above (GSE24206 padj = 3.16e-02, GSE32537 padj = 8.26e-17). Like periostin, collagen VI is an extracellular matrix protein which is involved in cellular adhesion [43]; it is also associated with integrin signaling. Differential splicing of COL6A3, and in particular of exon 4, has previously been associated with pancreatic cancer [44] and colon cancer [45], though in these studies it was inclusion as opposed to exclusion of exon 4 that associated with the disease phenotype. COL6A3 has been shown to be a target of the TGF-*β*/Smad signaling pathway [46], which is believed to play a role in the pathogenesis of idiopathic pulmonary fibrosis [4].

### IPF Gene Explorer

**Figure 10:**
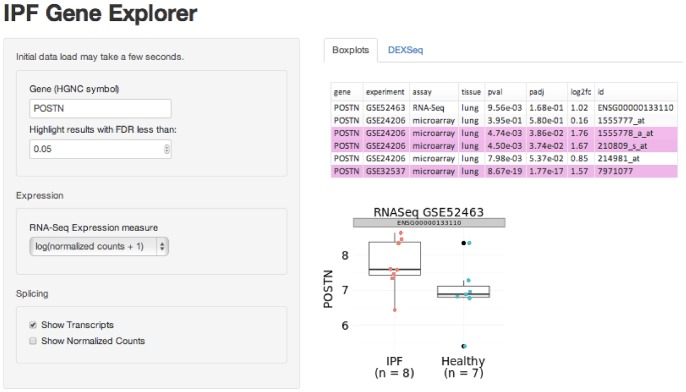
The IPF Gene Explorer displays expression data for a single user-selected gene.

**Figure 11:**
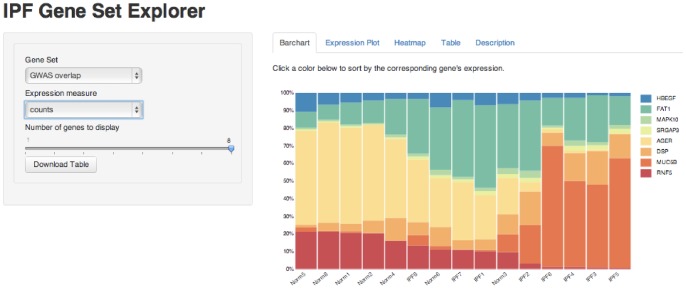
The IPF Gene Set Explorer displays expression data for a set of user-selected genes.

We created a web application built from the shiny package for R [47] to visualize the results of our gene expression study as well as previously published microarray studies. The website consists of two pages: one dedicated to viewing information about a single gene (Figure 10), and another for visualizing sets of genes (Figure 11), such as genes involved in a biological pathway or genes which are significantly differentially expressed.

The single gene page takes as input a gene by its HGNC gene symbol, and displays the p-values, adjusted p-values, and fold changes found in our study and in two recent microarray experiments [7], [8]. Boxplots of expression data are shown; for RNA-Seq data the user may choose to see counts, log-normalized counts, or variance stabilized expression data [6]. For the microarray experiments, expression data has been processed as described in the respective papers: briefly, log transformed data is normalized by Robust Multi-array Average (RMA), and in the case of GSE32537, expression values for probes in the same gene are averaged and a variance filter is employed [7], [8].

The gene set page provides a number of ways for visualizing the relative expression of genes across our 15 samples, including an interactive barchart, an expression plot, and a heatmap. The user may also view and download a table showing p-values, adjusted p-values, and log fold changes for the genes in the set.

## Discussion

Interrogation of transcription through RNA sequencing enabled us to discover genes whose differential expression reaches genome-wide significance in IPF, and to detect alternative splicing events which are not easily observed with traditional microarray experiments. We identified many differentially expressed genes and exonic regions, and validated two alternative splicing events by quantitative PCR. We believe these results indicate a potential role for alternative splicing of periostin and collagen VI alpha-3 in IPF, but more investigation is needed to determine the cell types in which alternative splicing is operative, and to identify causal variants and mechanisms of this effect and their relationship to IPF. We were not able to reproduce POSTN splicing results in a smaller sample of IPF lung fibroblasts (4 IPF samples and 4 healthy controls); this could indicate that the effect manifests primarily in a different cell type, or that we had insufficient power to detect the effect with this small sample size. We looked at SNPs from the 1000 Genomes CEU population (Utah residents with Northern European ancestry) lying within 50bp of periostin and COL6A3 splice sites, and found that none of these variants were significantly associated with IPF in the recent GWAS study [5]. Our results indicate that RNA-Seq has the potential to identify novel gene targets for further research, and larger studies and follow-up experiments will shed further light on the mechanisms underlying IPF. We believe that public access and easy visualization of results will enhance research efforts across disciplines in understanding this disease.

To our knowledge there has been only one published study examining gene expression in IPF lung tissues via RNA-Seq [48], which used samples from three IPF lungs and three COPD controls. Although this previous study did find differential splicing of periostin at an FDR of <10% (adjusted p-value = 0.0694), overall the set of genes which they find to have significant differential splicing at FDR <5% has small overlap with the set of genes having at least one differentially expressed exonic region (FDR<5%) in our data: only 7 genes are claimed to be significantly differentially spliced in both experiments, which is not more than expected by chance alone (1628 genes tested by both experiments, of which 147 are called significant here and 132 are called significant in [48]). There could be many reasons for this lack of replication. First, control samples are drawn from different clinical groups; second, [48] uses transcript quantification where we use differential exon usage, which introduces an extra layer of statistical modelling and estimation; third, both study sizes are relatively small, though our larger sample size should give us greater power to detect effects.

## Materials and Methods

Unprocessed fastq files and processed gene and exon counts are available at NCBI’s Sequence Read Archive (SRA) and NCBI’s Gene Expression Omnibus [49] through the GEO Series accession number GSE52463 (http://www.ncbi.nlm.nih.gov/geo/query/acc.cgi?acc=GSE52463). Scripts for downstream quantitative analyses are available at https://github.com/datapixie/ipf.

### IRB Protocols

Sample collection and research activities approved by University of Pittsburgh IRB protocol: IRB970946 and by Stanford IRB protocol: 18891 (Pathogenesis and progression of interstitial lung disease). Participants provided written consent to participate in study. The ethics committees approved the consent procedure. All data was analyzed anonymously.

### Human Lung Samples

**Figure 12:**
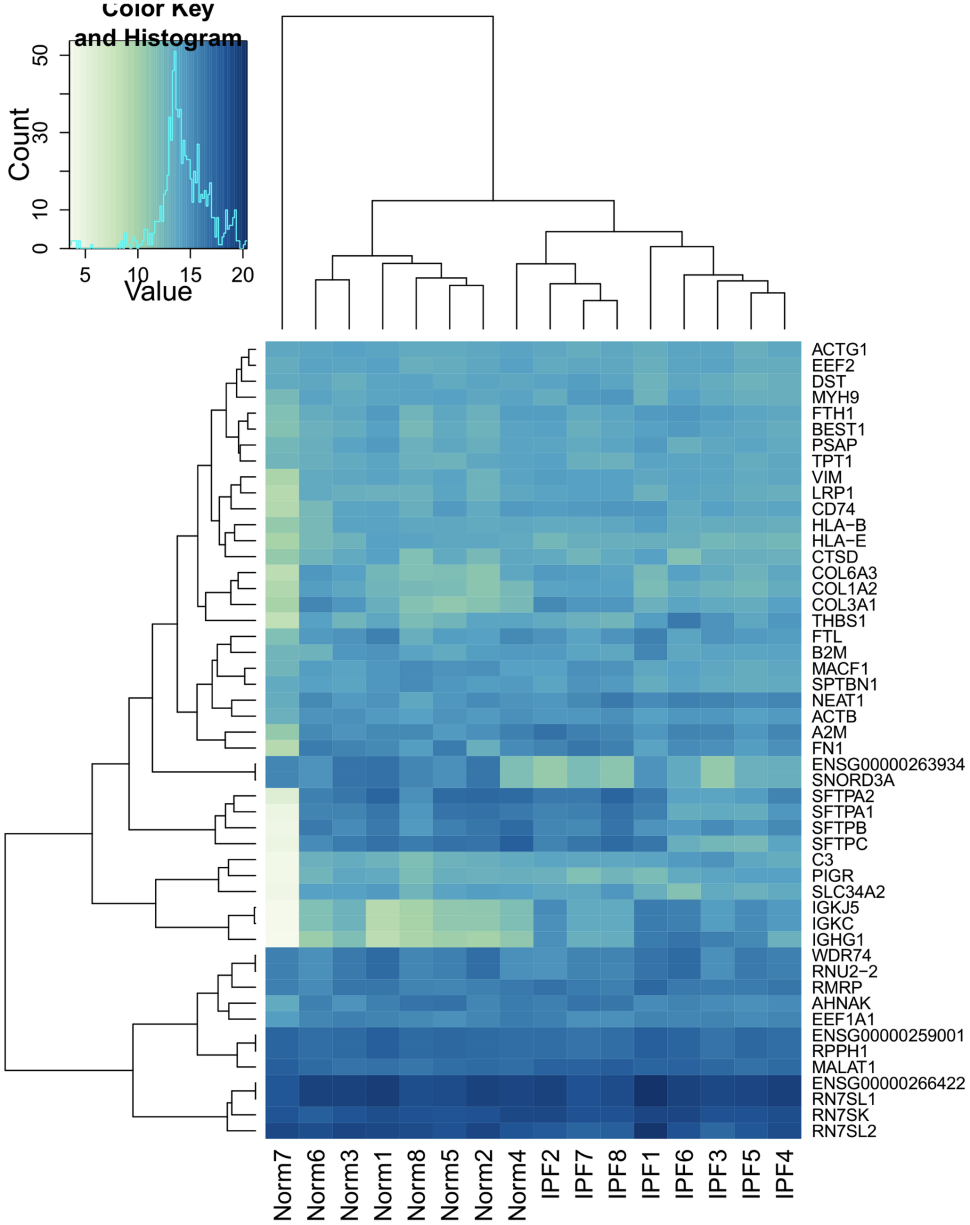
Norm 7 is an outlier in gene expression. This heatmap shows the top 50 most highly expressed genes (average across all samples) and corresponding hierarchical clustering of samples.

We obtained 8 healthy lung samples and 8 IPF lung samples from resources within Stanford University and the University of Pittsburgh. IPF tissue was obtained from patients undergoing surgical biopsy or transplantation for the diagnosis of interstitial lung disease or lung transplant for IPF. Control lung tissue was taken from patients undergoing lung cancer resection, remote from the cancer, or from donor explant lung which was not deemed suitable for transplantation. These samples were taken from heart-beating patients without prior flushing of the lungs. One healthy sample was determined to have extreme-outlier patterns of gene expression relative to the other 15 lung samples, and was excluded from downstream analyses (Figure 12). In particular, it showed extremely low expression of lung surfactant proteins SFTPA, SFTPC, and SFTPB (Table 8).

**Table 8:** Norm 7 shows outlier patterns of gene expression.

gene	Norm1	Norm2	Norm3	Norm4	Norm5	Norm6	Norm7	Norm8	IPF1	IPF2	IPF3	IPF4	IPF5	IPF6	IPF7	IPF8
SFTPB	70302	72895	42319	163757	42283	75872	14	21294	18673	38545	38986	40438	17076	21197	39943	110583
SFTPC	100497	77145	79640	270137	76353	35485	18	57248	51202	45706	5780	15055	3843	8924	81015	162671
SFTPA1	83001	87500	64306	97505	67908	53375	19	23898	36192	47494	11733	27762	7337	9621	55551	131945
SFTPA2	156777	126931	102191	176236	100685	59697	46	29664	49723	61079	14265	52364	14620	15679	70143	198983
PCSK1	16	13	32	49	10	56	19587	34	53	95	179	55	41	105	23	47
DIRAS3	13	23	11	27	8	29	25199	40	34	78	50	30	20	31	48	56
PCK1	0	2	2	2	14	77	26281	26	10	21	34	10	24	14	7	64
SCG2	12	8	44	19	9	29	32937	33	45	71	38	36	21	36	53	32
NEFL	0	3	2	1	7	14	39726	13	7	9	9	3	16	7	6	3
CHGA	2	2	17	9	12	18	63897	18	5	2	14	28	14	12	10	17

Normalized counts were variance-stabilized using the DESeq package to give “VST” expression values. Listed are the raw counts for genes which had VST <6 for one sample and average VST >14 for all other samples, or VST >14 in one sample and average VST <6 for all other samples. Healthy 7 was the only sample displaying such patterns. The four genes which are very highly expressed in all samples except Norm7 encode lung-specific surfactant proteins which reduce alveolar surface tension. Lack of these proteins in the lung leads to respiratory failure.

### Genotyping and Determination of Covariates

**Figure 13:**
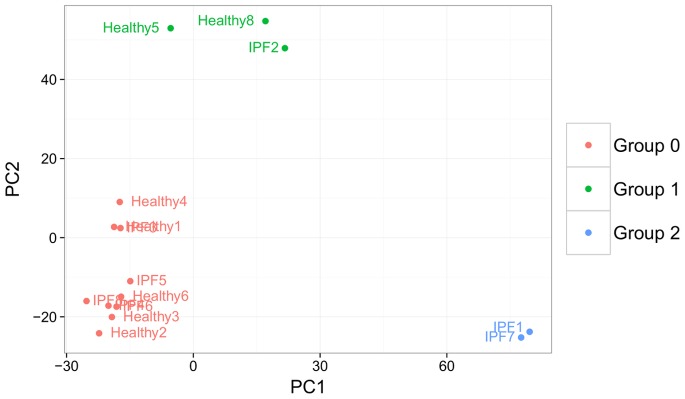
Subject genotypes cluster into three demographic groups. The first two principal components of sample genotype scores separate samples into three clusters.

**Figure 14:**
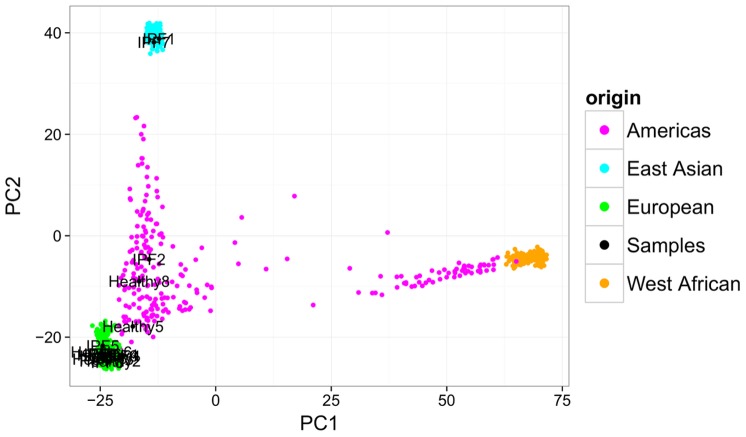
The three genotype clusters correspond to sample population-of-origin. The first two principal components for sample genotypes combined with corresponding Thousand Genomes genotypes are shown.

All samples were genotyped on Illumina Human Exome BeadChips, which give allelic information at >200,000 SNPs. Principal components (PC) analysis [50] was performed on these genotypes, with the first two PCs revealing three distinct clusters (Figure 13). Comparison to data from the Phase-One Thousand Genomes project [51] showed that this clustering corresponded to demographic structure among samples (Figure 14). We identified two IPF samples as coming from individuals of Asian descent and three samples from admixed or American descent, while the rest cluster with samples of European descent. The inclusion of each sample into one of these three demographic groups was used as a covariate in downstream differential expression analyses. We used Plink’s sex check tool (based on heterozygosity rates on the X chromosome [52]) to identify the sex of each sample, which was also used as a covariate.

### Library Preparation and Sequencing

RNA was isolated from all lung samples with Trizol and rRNA was depleted using the Ribo-Zero Magnetic Kit (Epicentre, Madison, WI). Briefly, 1 *μ*g total RNA was incubated with rRNA removal solution containing rRNA specific probes according to instructions at 68°C for 10 minutes. rRNA bound to probes was removed by magnetic bead pull-down. The final ribosomal depleted RNA was recovered following sodium acetate/glycogen addition and ethanol precipitation overnight. Samples were centrifuged at 10,000×g for 30 min, washed once per instruction, and resuspended with RNAse-free water. The remaining ribosomal depleted RNA was used to generate cDNA libraries using the Illumina TruSeq RNA preparation kit. Strand specificity was performed using dUTP during second strand synthesis. All samples were indexed with Illumina adapters and sequenced using an Illumina HiSeq 2000.

### Alignment and Counting of RNA-Seq Reads

Reads were aligned to the UCSC hg19 human reference genome using default parameters with STAR v2.0.2c [53], which was provided with splice junction information from Gencode v.14 annotation. The median number of reads for all 16 samples was about 26 million, and the median number of uniquely mapped reads was about 22.5 million (Table 9). Nonuniquely mapped reads and reads mapping to mitochondrial DNA were discarded. An in-house script was used to count reads over each gene in the gencode annotation, and DEXSeq-bundled scripts were used to count reads lying in non-overlapping exonic parts, as described in [54]. Read mappings were required to be properly stranded when performing these counts.

**Table 9:** Numbers of reads sequenced for each sample.

sample	number of reads	uniquely mapped reads
Norm1	20623466	17354393
Norm2	19711643	16939631
Norm3	23850384	21326596
Norm4	27568156	25210789
Norm5	21328567	18264192
Norm6	26054686	22831687
Norm7	29606940	26957981
Norm8	27182913	23959608
IPF1	21822005	18861962
IPF2	26696309	23524193
IPF3	28104723	24908240
IPF4	26197318	23168922
IPF5	23401239	20251950
IPF6	26780342	22191034
IPF7	23413788	20835580
IPF8	29640467	25248566

### Differential Expression Analysis

**Figure 15:**
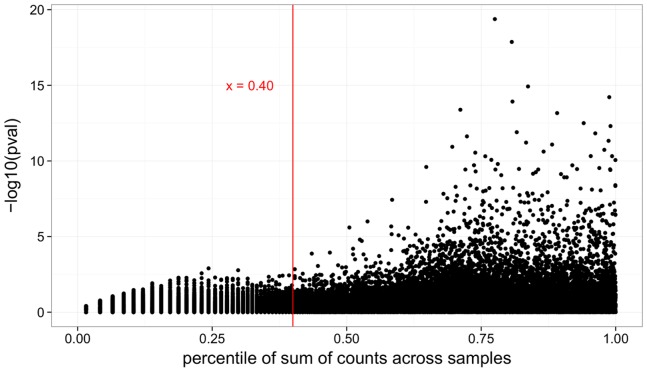
Genes with low read count are filtered prior to testing for differential expression. Each point represents a gene. We are able to increase our detection power by eliminating tests which have read counts below the 40th percentile, and therefore would have small probability of attaining significance [6].

The bioconductor package DESeq tests for differential gene expression by modeling read counts with a negative binomial distribution, where means are predicted via a generalized linear model (GLM) with logarithmic link and dependencies of variance on mean are estimated from the data. DESeq tests for differential expression by comparing the fit of the GLM with disease-state coefficient to one without, via a *χ* ^2^ likelihood-ratio test [6]. After filtering out the 40% lowest-expressed genes (Figure 15), we used DESeq v.1.10.1 to identify genes that were differentially expressed between IPF samples and healthy controls. The “pooled-CR” option was used to estimate dispersions and resulting p-values were adjusted by the method of Benjamini-Hochberg (BH) to account for multiple testing [55]. The related package DEXSeq v.1.0.2 [33] was used to find differential exon usage between the two cohorts. Overlapping genes were excluded from the differential exon analysis. Demographic group and sex were added as covariates in the generalized linear models used by both DESeq and DEXSeq.

### Microarray Comparison

We compared differentially expressed genes found in the RNA-Seq data with two recently published studies which used microarrays to estimate relative transcript expression [7], [8]. The data for these studies are publicly available on the Gene Expression Omnibus (GEO) website (accession IDs GSE24206 and GSE32537) [49]. Differential expression analysis was performed using the limma package [56], [57], via R scripts produced by GEO’s geo2R application.

### Gene Network Analysis

We determined pathway enrichment for differentially expressed or spliced genes by a method using p-value enrichment permutation analysis [58]. Gene sets were obtained from the following online databases: BioCarta (www.biocarta.com), KEGG (www.genome.jp/kegg), Pathway Interaction Database (pid.nci.nih.gov), Reactome (www.reactome.org), SigmaAldrich (www.sigmaaldrich.com/life-science.html), Signaling Gateway (www.signaling-gateway.org), Signal Transduction KE (stke.sciencemag.org), SuperArray (www.superarray.com). A score for each gene (resp., exon) was computed as the negative log_10_ of the p-value output from DESeq, and the median of scores in each gene set was used as the gene set’s score. Gene (exon) scores were permuted so as to preserve the number of genes (exons) in each pathway and the number of pathways corresponding to each gene; gene set scores were then recomputed, and this was repeated 10,000 times. The distribution of scores for each gene set was used to establish an empirical p-value, and p-values for gene sets were adjusted by BH.

We compared these results with those from SPIA [32], a Bioconductor network analysis package which incorporates knowledge of network topology and gene expression fold change to determine if a pathway is activated or inactivated in IPF relative to healthy samples. We provided SPIA with the fold changes of genes which were found to have significant differential expression at 1%, 5%, and 10% FDR. Because SPIA utilizes information at the gene-level, this analysis could not be used to identify pathways enriched for alternative splicing events.

### GWAS Enrichment

In order to determine whether the overlap between the set of differentially expressed genes and the set of GWAS-identified genes (both considered as subsets of those genes tested for differential expression) is larger than expected by chance alone, we calculated a p-value of 0.013 from the hypergeometric distribution in R as:
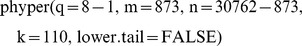
where q is the size of the overlap minus one, m is the number of genes found to be significantly differentially expressed at FDR 5%, n is the number of ensembl IDs tested for differential expression which were not called significant, and k is the number of GWAS-identified genes which were tested for differential expression. This p-value is equal to the probability of drawing 8 or more green balls from an urn containing 873 green balls and 29889 red balls, when you have drawn a total of 110 balls randomly from the urn. Since the p-value indicates that it is unlikely to see an intersection of 8 genes by chance alone, we conclude that the GWAS-identified genes are enriched for differential expression. Other hypergeometric p-values were calculated similarly.

For the sample-based permutation approach, we permuted the disease status (IPF or healthy) of our 15 subjects. For each permutation, DESeq was rerun and the size of overlap between significant genes and GWAS genes was computed. We ran 200 permutations, and of these only two had overlaps of size 8 or larger, giving an empirical p-value of 0.01.

### PCR Validation

POSTN primers for exons 20 and 21 were designed to produce amplicons of approximately the same length that were solely contained within each respective exon at final concentrations of 500nM (POSTN 20FWD-2: ACTAGGATTTCTACTGGAGGTGGA; POSTN 20REV-2: ACAATTTCTTCAGAGTTTCTTCTGT; POSTN 21 FWD-2: AGGTCACCAAGGTCACCAAA; POSTN 21REV-3 TCAAATAAATGACCATCACCACCT). Phusion High-Fidelity Polymerase (0.02U/ul) and 1x buffer (NEB, Ipswich, MA) were used with a final concentration of 0.6x SYBR Green (Molecular Probes, Eugene OR) and 0.2mM dNTPs for 40 cycles on a StepOnePlus Real-Time Q-PCR machine (Life Technologies, Carlsbad, CA). cDNA libraries used for RNA-Seq representing healthy and IPF lung samples were diluted over 5 two-fold dilutions for Q-PCR and amplified to produce standard curves.

Real time PCR was performed for each sample and each of the two primers at five different dilutions. For each sample, the machine-calculated optimal threshold was found for each exon primer and concentration separately, and *C_t_* values were calculated by using the mean of these thresholds for both exons. We then calculated standard curves for each (sample, exon) pair as a quality control to confirm linearity between *C_t_* and log concentration. The expression ratio for each sample was calculated as
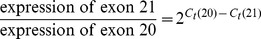
where *C_t_*(*j*) is the *C_t_* value found for exon *j* at the most concentrated dilution (0.1).

PCR validation for COL6A3 was performed similarly (primers COL6A3 E057-FWD: CCGATATTGGCCAACTCCCC; COL6A3 E057-REV: GACACCTACTCCACCAAGGC; COL6A3 E050-FWD: ATGAGGGTGCGAACGTACTG; COL6A3 E050-REV: GCAAGAGGGACGTGGTCTTT).
