# Correction: Transcriptome Analysis Reveals Differential Splicing Events in IPF Lung Tissue

**DOI:** 10.1371/journal.pone.0097550

**Published:** 2014-05-07

**Authors:** The PLOS ONE Staff

## Notice of Republication

This article was republished on April 21, 2014, to correct the figure order. Please download the article again to view the correct version. The originally published, uncorrected article and the republished, corrected article are provided here for reference.

## Supporting Information

File S1 Originally published, uncorrected article.
(PDF)

File S2 Republished, corrected article.
(PDF)

## References

[1] Nance T, Smith KS, Anaya V, Richardson R, Ho L, et al (2014) Transcriptome Analysis Reveals Differential Splicing Events in IPF Lung Tissue. PLoS ONE 9(3): e92111 doi:10.1371/journal.pone.0092111 24647608 10.1371/journal.pone.0092111 PMC3960165

